# Rituximab, lenalidomide and BTK inhibitor as frontline treatment for elderly or unfit patients with diffuse large B-cell lymphoma: a real-world analysis of single center

**DOI:** 10.1186/s40164-022-00314-w

**Published:** 2022-09-16

**Authors:** Yanan Zhu, Xiang Zhang, Juying Wei, Chunmei Yang, Hongyan Tong, Wenyuan Mai, Min Yang, Jiejing Qian, Liping Mao, Haitao Meng, Jie Jin, Wenjuan Yu

**Affiliations:** 1 Department of Hematology, The First Affiliated Hospital, Zhejiang University School of Medicine, Hangzhou, Zhejiang People’s Republic of China; 2 Zhejiang Provincial Key Laboratory of Hematopoietic Malignancy, Zhejiang University, Hangzhou, Zhejiang People’s Republic of China; 3 Zhejiang Provincial Clinical Research Center for Hematological Disorders, Hangzhou, Zhejiang, People’s Republic of China; 4 Zhejiang University Cancer Center, Hangzhou, Zhejiang People’s Republic of China

## Abstract

The combination of rituximab, lenalidomide, and Bruton’s tyrosine kinase inhibitor (BTKi) ibrutinib, followed by chemotherapy, has shown high efficacy in patients with newly diagnosed diffuse large B-cell lymphoma (DLBCL) in Smart Start trial. We aimed to evaluate the efficacy, safety of SMART (rituximab + lenalidomide + BTKi) regimen and SMART–START regimen as a first-line treatment in elderly or unfit DLBCL patients. 31 patients were included, 17 used SMART regimen, with median age 82 years, 14 unfit patients received SMART–START regimen. 14/16 (87.5%) patients in SMART group achieved overall response (OR), with 10/16 (62.5%) achieved complete response (CR). 12/13 (92.3%) patients in SMART–START group achieved OR, with 8/13 (61.5%) achieved CR. With a median follow-up of 15.4 (3–29.1) months, median progression-free survival (PFS) and overall survival (OS) have not been reached, 1-year PFS was 81% in SMART group and 84% in SMART–START group. Common grade 3–4 adverse events (AEs) during SMART regimen were neutropenia (8 [25.8%]), infection (6 [19.4%]) and skin rash (3 [9.7%]). Our study shows that SMART regimen is an effective and safe therapy for elderly DLBCL patients, and SMART–START regimen can be used in unfit patients who could not tolerate intensive chemotherapy in the onset.

**Supplementary Information:**

The online version contains supplementary material available at 10.1186/s40164-022-00314-w.

## Letter to the editor

Diffuse large B-cell lymphoma is the most common lymphoma subtype of non-Hodgkin lymphoma (NHL), and its incidence increases with ag [1]. Although there are some studies showing that R-CHOP can be used in elder DLBCL patients [2, 3], some other studies have shown that comorbidities and organ function impairment may lead to unmanageable toxicities, and thus limit chemotherapy dosage [4, 5]. Furthermore, DLBCL in elderly patients may be biologically different from DLBCL in younger patients, there exists more non-germinal center B-like (non-GCB) subtype in elderly patients [6]. In recent years, more and more non-cytotoxic agents were applied to DLBCL patients, including the BTK inhibitors, lenalidomide. Although both ibrutinib and lenalidomide could not significantly improve outcomes in DLBCL when added to R-CHOP as single agent [7–9], the combination of ibrutinib and lenalidomide has a synthetic lethality against DLBCL [10]. Westin et al. demonstrated that chemotherapy-free combination of rituximab, ibrutinib, lenalidomide was highly effective in patients with newly diagnosed non-GCB DLBCL, in Smart Start trial [11]. Goy et al. reported the triplet of ibrutinib, lenalidomide, and rituximab demonstrated promising activity in patients with relapsed/refractory DLBCL, particularly non-GCB DLBCL [12].

Depend on the inspiring outcomes of these previous studies, our center tried to use SMART regimen as a first-line therapy in elderly patients with newly diagnosed DLBCL, and SMART–START regimen in patients with comorbidities and poor performance status, who had decreased tolerance to intensive chemotherapy in the onset of disease. We aimed to assess the efficacy and safety of the SMART and SMART–START regimen in a real-world patient group.

31 patients were commenced from October 2019 to November 2021 in our center. In SMART regimen group, elderly patients received SMART regimen, which means combination of rituximab (d1, 375 mg/m^2^ intravenously), lenalidomide (25 mg orally d2–15) and BTK inhibitor (ibrutinib 560 mg orally daily or zanubrutinib 160 mg twice daily or orelabrutinib 150 mg daily), 21-day a cycle for 6–8 cycles. In SMART–START group, unfit DLBCL patients who couldn’t tolerate intensive chemotherapy in the onset received SMART regimen for the first 2–3 cycles, and then added with chemotherapy. 58% were male; Median age was 75 (56–93), the average age is older in SMART group, which is 82 (73–93), compared with 67 (56–78) in the SMART–START group. Patients with poor performance status (PS) at diagnosis was higher in SAMRT–START group, 9/14 (64.3%) had Eastern Cooperative Oncology Group (ECOG) PS 2–4. 64% had advanced stage (III/IV), 23% had B symptoms and 29% had bulky disease. All patients in SMART group were non-GCB type while half of the patients in SMART–START group were GCB type. In the SMART group, 6 patients received ibrutinib, 9 used zanubrutinib, 2 used orelabrutinib. In SMART–START group, 10 patients used ibrutinib, 2 used zanubrutinib, 2 used orelabrutinib. In terms of chemotherapy, 7 received RCHOP, 3 received R-DAEPOCH, due to their immunohistochemistry results showed double express, 3 received R-Gemox/RminiCHOP, 1 was 78 years old, 1 had hepatitis B virus-related cirrhosis with grade 3 thrombocytopenia, 1 had heart involved, with history of subtotal gastrectomy. For comprehensive baseline characteristics see Table 1.

**Table 1:** Baseline characteristics

Characteristic	Whole cohort	SMART	SMART–START
	N = 31	N = 17	N = 14
Median age (range)	75 (56–93)	82 (73–93)	67 (56–78)
< 75, n (%)	13 (41.9%)	1 (5.9%)	12 (85.7%)
≥ 75, n (%)	18 (58.1%)	16 (94.1%)	2 (14.3%)
Male sex, n (%)	18 (58.1%%)	8 (47.1%)	10 (71.4%)
ECOG ≥ 2, n (%)	17 (54.8%)	8 (47.1%)	9 (64.3%)
Stage, n (%)			
I–II	11 (35.5%)	7 (41.2%)	4 (28.6%)
III–IV	20 (64.5%)	10 (58.8%)	10 (71.4%)
No. EN sites, n (%)			
< 2	17 (54.8%)	11 (64.7%)	6 (42.9%)
≥ 2	14 (45.2%)	6 (35.3%)	8 (57.1%)
Elevated S-LDH, n (%)	19 (61.3%)	11 (64.7%)	8 (57.1%)
IPI, n (%)			
0–2	11 (35.5%)	7 (41.2%)	4 (28.6%)
3–5	20 (64.5%)	10 (58.8%)	10 (71.4%)
B-symptoms, n (%)	7 (22.6%)	4 (23.5%)	3 (21.4%)
Bulky disease ≥ 7.5 cm, n (%)	9 (29.0%)	6 (35.3%)	3 (21.4%)
Cell of origin, n (%)			
GCB	7 (22.6%)	0 (0%)	7 (50%)
Non-GCB	24 (77.4%)	17 (100%)	7 (50%)

*ECOG* Eastern Cooperative Oncology Group, *EN* extranodal. *IPI* international prognostic index, *LDH* lactate dehydrogenase, *GCB* germinal center B-like, *non-GCB* non-germinal center B-like

The ORR in SMART group was 87.5% (14/16), with 62.5% (10/16) achieving CR, and 25% (4/16) achieving partial response (PR). 1 patient had no measurable lesion, he discontinued therapy after two cycles of smart regimen due to personal choice, and this patient is still alive after stopping therapy for 1.5 years. The median cycle of SMART regimen was 5.2, median time to response was 1.2 (0.6–2.9) months, median time to best response was 3.1 (0.7–6) months. The ORR in SMART–START group was 92.3% (12/13), with CR 61.5% (8/13) and PR 30.7% (4/13). Before chemotherapy, 3/13 (23.1%) patients achieved CR, 8/13 (61.5%) patients achieved PR, 1 patient was stable disease (SD) and 1 patient had progressive disease (PD). The patient with SD after 2 cycles of IR2 changed to IR2-DAEPOCH and achieved CR.

**Fig. 1:**
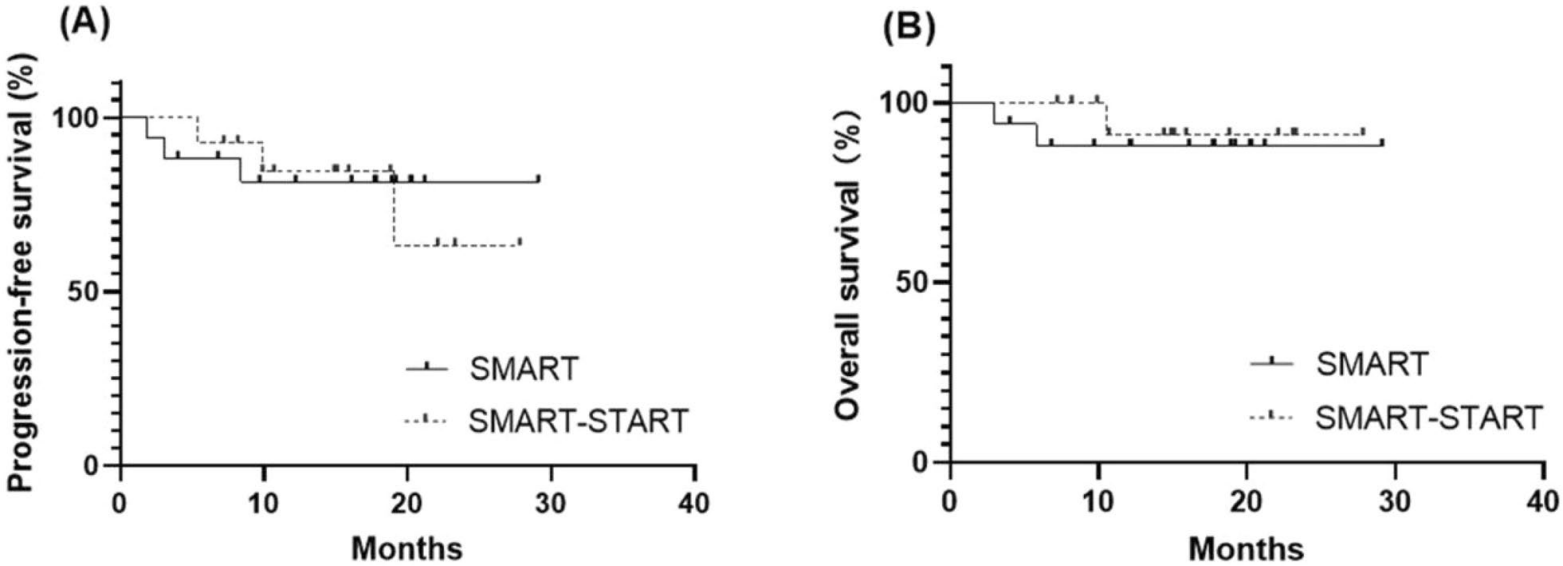
Survival outcomes after a median follow-up of 15.4 months. **A** Median progression-free survival time in all patients was not reached, 1-year PFS was 81% in SMART group and 84% in SMART–START group. **B** Median overall survival time in all patients was not reached, 1-year overall survival was 89% in SMART group and 91% in SMART–START group

With a median follow-up of 15.4 months (3–29.1 months), median PFS and OS have not been reached,1-year PFS was 81% in SMART group and 84% in SMART–START group; 1-year OS was 89% in SMART group and 91% in SMART–START group. 2 patients in SMART group and 1 in SMART–START group died at data lock, all due to progressive disease. Kaplan–Meier analyses of PFS and OS of the two groups are displayed in Fig. 1.

Grade 3–4 adverse events during chemo-free treatment are displayed in Additional file 1: Table S1. The most common grade 3–4 AE was neutropenia (8/31). 6 patients had infections: 2 pneumonia, of whom 1 was pulmonary fungal infection, 2 febrile neutropenia, 1 urinary tract infection, 1 herpes zoster. Skin rash occurred in 11 patients, of whom 3 were grade 3–4, and 2 of them had lenalidomide interruption, 1 had lenalidomide dose reduction.

Our study showed SMART regimen was a good option for the first line treatment of elderly DLBCL patients, which was well tolerated and had promising efficacy. SMART–START regimen was also safe and effective in unfit patients who were not considered to tolerate standard RCHOP in the onset. However, this study is a retrospective study in single center, the number of enrolled patients was small, and median follow-up time was only 15.4 months, so there might be some bias in the assessment of response and toxicities. Further randomized studies are needed to get adequate evidence.

## Supplementary Information

**Additional file 1: Table S1.** Adverse events grade 3–4.

## Data Availability

The data in the current study are not public, but it can be obtained from the authors upon request.

## Abbreviations

BTKi: Bruton’s tyrosine kinase inhibitor
DLBCL: Diffuse large B-cell lymphoma
OR: Overall response
CR: Complete response
PFS: Progression-free survival
OS: Overall survival
AE: Adverse event
NHL: Non-Hodgkin lymphoma
GCB: Germinal center B-like
PS: Performance status
ECOG: Eastern Cooperative Oncology Group
PR: Partial response
SD: Stable disease
PD: Progressive disease
