# Supplementary material for: Rituximab, lenalidomide and BTK inhibitor as frontline treatment for elderly or unfit patients with diffuse large B-cell lymphoma: a real-world analysis of single center
Source: Exp Hematol Oncol. 2022 Sep 16;11:57. doi: 10.1186/s40164-022-00314-w (PMC9479281; doi:10.1186/s40164-022-00314-w)
Supplement: Supplementary file 1 — Additional file 1: Table S1. Adverse events grade 3–4. [file 40164_2022_314_MOESM1_ESM.docx]

Table S1: Adverse events grade 3-4

| Adverse events | Grade 3-4, n (%) |
| --- | --- |
| Hematologic AEs |  |
| Neutropenia | 8 (25.8) |
| Anemia | 0 (0.0) |
| Thrombocytopenia | 2 (6.5) |
| Infections |  |
| Pneumonia | 2 (6.5) |
| Febrile neutropenia or septicemia | 2 (6.5) |
| Other^1^ | 2 (6.5) |
| Other Non-hematologic AEs |  |
| Skin rash | 3 (9.7) |
| Edema | 2 (6.5) |
| Fatigue | 1 (3.2) |
| Decreased appetite | 1 (3.2) |

AE: adverse event; ^1^1 patient had urinary tract infection, 1 patient had herpes zoster.
